# Adherence to dietary recommendation and associated factors among diabetic patients in Ethiopian teaching hospitals

**DOI:** 10.11604/pamj.2019.33.260.14463

**Published:** 2019-07-26

**Authors:** Mohammed Akibu Mohammed, Nigussie Tadesse Sharew

**Affiliations:** 1 Debre Berhan University, Institute of Medicine and Health science, Department of Midwifery Debre Berhan, Ethiopia

**Keywords:** Adherence, diabetic patients, dietary recommendation, Ethiopia, teaching hospital

## Abstract

**Introduction:**

Dietary management is considered to be one of the cornerstones of diabetes care. Improvement of dietary practice alone can reduce glycosylated hemoglobin (HbA1c) by an absolute 1 to 2% with the greatest impact at the initial stages of diabetes.

**Methods:**

Data from Hospital based cross sectional study were used to assess the level of dietary adherence and its determinants among diabetic patients. The morisky 8 item medication adherence scale was used to develop 10 item tool for evaluation of dietary adherence. Multiple logistic regression was conducted to identify factors which affect dietary adherence and variables with P vale < 0.05 were considered statistically significant.

**Results:**

More than half of 303 participants (55.7%) were found to be non-adherent to the recommended dietary approach. Gathering with family and friends and eating out were the major reasons for not being compliant with the recommended regimen. Attending diabetic nutrition education (AOR=2.8 95% C 1.97, 5.61) and having the disease for more than 10 years (AOR 2.9 95% CI 1.32, 5.84) were statistically significant with adherence to dietary recommendation.

**Conclusion:**

Non-adherence to recommended dietary practice was observed in more than fifty percent of patients; it is therefore a major public health problem. Attending diabetic nutrition education and length of diabetes greater than 10 years were the factors associated with adherence to dietary recommendation. This findings indicate that it is important to design strategies to help patients understand their dietary regimens and improve their adherence.

## Introduction

Diabetes is one of the most rapidly increasing noncommunicable diseases and an important public health problem all over the world [1]. The global burden of diabetes is rising dramatically worldwide and an estimated 422 million adults are currently living with diabetes mellitus [2, 3]. As such, a previous estimate that the diabetes prevalence would increase from 171 to 276 million between 2000 and 2030 has already been exceeded. A more recent estimate suggests that the prevalence will reach 642 million people in 2040 [4]. Diabetes is unevenly distributed with over 70% of people with diabetes reside in low- and middle-income countries particularly sub-Saharan Africa, a region already heavily burdened by communicable disease [5]. Similarly diabetes has been a major public health concern in Ethiopia as the number of deaths attributed to diabetes reached over 21,000 in 2007 and this estimate has increased to about 25,000 and 34262 in 2011 and 2013 respectively [6, 7]. Management of the disorder creates a great physical, psychological and socioeconomic burden on families and society, thus, prevention with diet and lifestyle modifications should be prioritized [8]. Glycemic control is achieved by undertaking and sustaining a complex array of self-care behaviors, including four main domains: taking medications, sustaining appropriate dietary practice, engaging in regular exercise and self-monitoring of blood glucose levels [9, 10]. Of the preferred life style modifications for diabetes management, dietary modification is considered to be one of the cornerstones of diabetes care. Appropriate dietary practices emphasizes the intake of less fat, more fiber, less sodium and more foods that have health-promoting properties such as fish, soy products, fruits and vegetable [11, 12]. Improvement of dietary practice alone can reduce glycosylated hemoglobin (HbA1c) by an absolute 1 to 2% with the greatest impact at the initial stages of diabetes; and its effects are apparent after 6 to 12 weeks of initiation [13]. Various studies have been conducted to assess adherence to an appropriate dietary regimen as part of diabetes self-management. Unfortunately, most of these studies indicate a low level of adherence to the recommended dietary regimen [14]. For example, a study in India showed that dietary prescriptions were followed regularly only by 37% of patients [15]. Similarly, studies conducted in Jimma and Addis Ababa, Ethiopia has also revealed that the level of diabetic self-care practice was insufficient among study participants [16, 17]. In Ethiopia, the paucity of health information and standard guideline related to diet has been posing crises on long-term glycemic control in diabetes patients. At the same time, care givers often give less attention to describe dietary recommendation and their appropriate preparation. Therefore, this study assesses the level of adherence to the recommended dietary practices and its associated factors among diabetic patients in selected Ethiopian Teaching Hospitals located in-Addis Ababa, Ethiopia.

## Methods

**Study setting:** the study was conducted in the Diabetic Outpatient Department (OPD) of three selected teaching hospitals (Tirunesh Beijing, black lion specialized and Saint Paul specialized hospitals) which are located in Addis Ababa, the capital city of Ethiopia. The data were collected from November 2016 to February 2017.

**Study design and population**: data for this analysis are from a Hospital based cross sectional study. These date are used to assess the rate of adherence to dietary recommendations and its predictors among diabetic patients. All registered Diabetic patients who have been attending a diabetic clinic for follow-up were included with the exception of patients who are under age 18, pregnant, or critically ill and patients whose diagnosis occurred less than 6 months prior.

**Sample size and sampling procedure**: the sample size (326) was calculated using a single population proportion formula; given the prevalence diabetic treatment adherence was 67% [18], Confidence Interval of 95%, 5% Marginal error and 10% non-respondent rate. Systematic random sampling with kth value of 4 was used to select the study subjects.

**Data collection and quality control:** data were collected using a structured questionnaire administered via face to face interview. Dietary recommendations comprised of a recommendation by a health care professional of a diet comprising of High fiber diet, fruits and vegetables (at least 5 servings per day), eat very few sweets, low-fat milk and dairy products. The Morisky 8 item Medication Adherence Scale was used to develop a 10-items tool measuring the level of adherence to dietary recommendations. Each of the items contained two response options (Yes = 1 and No= 0). Pre-tests were administered with five percent of the total sample size and necessary adjustments were made. The questionnaire was also tested for internal consistency (reliability) with Cronbach's Alpha test (0.74). The completeness, consistency, and accuracy of the collected data were examined by principal investigators every day.

### Operational definition

**Adherent:** a value below the mean score (score of 5.81) on 10 item dietary adherence assessment scale).

**Non adherent:** a score at or above the mean value on 10 item dietary adherence assessment scale.

**Data processing and analysis:** the data were coded, cleaned and entered into EPI-INFO version 7 and exported into SPSS version 20 for statistical analysis. First, descriptive statistics were generated. Then, binary and multiple logistic regressions were used to examine the possible association between the determinant and the outcome variable. In the model, P-value < 0.05 was used to indicate a statistically significant association.

**Ethical consideration:** this study was approved by the ethical review committee of the Institute of Medicine and Health Science, Debre Berhan University. An official letter of permission was provided to the administrative office of each hospital. The respondents were informed about the purpose of the study and written informed consent was obtained from each study participants. Information obtained was kept anonymous.

## Results

**Socio-demographic characteristics:** a total of 303 patients agreed to participate in the study, reflecting a response rate of 93%. Nearly half (47.9%) of participants were male. The mean age was 51 (± 7.6 yrs.), the majority of whom (51.5%) were between ages 41 and 60. Educational status of participants revealed that 43 (14.5%) were bachelor degree holders (Table 1).

**Table 1:** Socio-demographic characteristics study participants in Ethiopian teaching hospital, Ethiopia, 2017

Variables	Frequency	Percent
Sex		
Male	145	47.9
Female	158	52.1
Age category		
<40	111	36.6
41-60	156	51.5
>61	36	11.9
Marital status		
Unmarried	50	16.5
Married	197	65
Divorced	40	13.2
Widowed	16	5.3
Ethnicity		
Oromo	105	34.5
Amhara	84	27.7
Tigre	64	21.1
Gurage	39	12.9
Others^a^	11	3.6
Religion		
Orthodox	143	47.2
Muslim	88	29
Protestant	62	20.5
Catholic	8	2.6
Other^b^	2	0.7
Income		
<500 ETB	54	17.8
500–999 ETB	67	22.1
≥1000 ETB	182	60.1
Educational Status		
Illiterate	83	27.4
Primary Education	49	16.2
Secondary education	89	29.4
Diploma	39	12.9
University degree and Above	43	14.5
Occupation		
Government employee	96	31.7
Merchant	57	18.8
House wife	65	21.4
Student	41	13.6
Farmer	36	11.9
Other ^c^	8	2.6

a Afar, wolayita and Harari

b Woke-feta and baha-olla

c Daily laborer and jobless

**Health-related characteristics**: more than two-thirds (71.3%) of study participants were diagnosed with type 2 diabetes mellitus. The mean number of years since diagnosis was 5.09 (SD = 4.18) years, ranging from less than five years (58.8%) to more than ten years (11.5%). In 27.4% of patients, some other comorbid disease was identified from their medical record. About two in ten (22.8%) patients were found be obese. The majority of patients (62.3%) stated that they have received a well-organized diabetes nutritional education (Table 2).

**Table 2:** Health related characteristics among diabetes patients, in Ethiopian teaching hospitals, Ethiopia, 2017

Variables	Frequency	Percent
**Year of diagnosis**		
< 5 years	178	58.8
5– 10 years	90	29.7
>10 years	35	11.5
**Type of DM**		
Type 1 DM	87	28.7
Type 2 DM	216	71.3
**Medical Co morbidities**		
Hypertension	36	11.9
HIV infection	28	9.2
Dyslipidemia	13	4.3
Renal disease	6	2
No Comorbid illness	220	72.6
**BMI value**		
< 18.5 (Underweight)	16	5.3
18.5 – 24.5 (Normal weight)	143	47.2
25 -29.5 (Over weight)	75	24.7
≥ 30 (Obesity)	69	22.8
**Diabetes Nutritional education**		
Received	187	62.3
Not received	114	37.7

**Adherence to dietary recommendation and barriers to adherence:** the overall magnitude of adherence to dietary recommendation was 134 (44.3%) (95% CI, 38.4, 51.74) among diabetic patients participated in the study Table 3. Most of the diabetic patients (38.7%) believed that a high frequency of food gatherings with family and friends affected their healthy dietary plan and 21.8% of participants said that factors like eating out at restaurants and inappropriate food offers by others interfere with their healthy dietary plan (Table 4).

**Table 3:** Morisky medication adherence questionnaire for assessing the level of adherence to Dietary recommendation among diabetes patients, in Ethiopian teaching hospital, Ethiopia, 2017

Components	Frequency	Percentage (%)
**Do you sometimes forget to follow the recommended dietary approach For DM?**		
Yes	190	62.7
No	113	37.3
**Over the past two weeks, were there any days when you did not take your dietary plan properly?**		
Yes	146	48.1
No	157	51.9
**Did you miss the proper dietary plan yesterday?**		
Yes	127	41.9
No	176	58.1
**Have you ever cut back or stopped following the recommended dietary plan without telling your doctor because you felt unnecessary to do so?**		
Yes	164	54.2
No	139	45.8
**When you feel like your DM is under control, do you sometimes stop taking your dietary plan?**		
Yes	148	48.7
No	155	51.3
**When you travel or leave home, do you sometimes forced to stop following your dietary plan?**		
Yes	188	62.1
No	115	37.9
**Do you ever feel hassled about sticking to your dietary plan?**		
Yes	186	61.3
No	117	38.7
**Did you have feelings of dietary deprivation?**		
Yes	227	74.9
No	76	25.1
**Do you forget to include fruits and vegetables in your dietary plan**		
Yes	208	68.7
No	95	31.3
**Do you forget to cut down butter and fat intake in your food**		
Yes	175	57.8
No	128	42.2

**Table 4:** Barriers for non-adherence with the recommended dietary regimen

Reasons	Frequency	Percent
Inappropriate dietary habits (e.g. Eating snacks in-between meals)	11	6.6
Financial constraints	33	19.2
Gathering with friends and family	65	38.7
Eating outside home	37	21.8
It takes efforts	30	17.8
Being busy	24	14.3
Costs extra many	27	16.3

**Factors associated with adherence to dietary recommendations:** in a multivariate logistic regression model, patients who have received diabetic nutrition education were nearly three times (AOR= 2.8, 95% CI 1.97, 5.61) more likely to adhere to the recommended dietary practices than patients without nutritional education. In addition, the odds of following the recommended dietary plan were three times (AOR 2.9 95% CI 1.32, 5.84) higher among patients whose diagnosis was received more than ten years ago (Table 5).

**Table 5:** Factors associated with adherence to the recommended dietary regime

Variable	Dietary Adherence		COR (95%, CI)	AOR (95%, CI)
	Adherent	Non- Adherent		
**Educational status**				
**Elementary**	17 (34.1)	32 (65.3)	2.23 (0.73-4.4)	4.48 (0.87-6.9)
**High school**	24 (27)	65 (73)	1.67 (1.2-1.8)	1.28 (.85-5.9)
**Diploma**	27 (64.3)	16 (35.7)	2.74 (0.9-8.41)	2.21 (0.76-6.22)
**Degree and above**	37 (89.7)	2 (10.3)	4.98 (3.6-9.3)	2.74 (0.46-7.28)
**No education**	29 (34.9)	54 (65.1)	1	1
Diabetic Nutrition Education				
**Received education**	103 (54.5)	86 (45.5)	3.2 (2.1 – 7.74)*	2.8 (1.97 -5.61)*
**Not received education**	31 (27.1)	83 (72.9)	1	1
Type of DM				
**Type 2 DM**	82(38.9)	134 (61.1)	0.41 (.27-.92)*	0.57 (.42 -1.38)
**Type 1 DM**	52 (59.8)	35 (40.2)	1	1
Medical Comorbidity				
**Present**	57 (68.7)	26 (31.3)	4.07 (2.58 -7.21)*	1.95 (0.84 – 3.18)
**Absent**	77 (35)	143 (65)	1	1
Duration since Diagnosis				
**Over 10 years**	22 (62.9)	13 (37.1)	3.4 (1.78 – 7.81)	2.9 (1.32 – 5.84)*
**5 to 10 years**	36 (40)	54 (60)	2.7 (0.78- 4.59)	3.3 (0.94 – 6.42)
**Less than 5 years**	82 (46)	96 (54)	1	1

* **P-value** < 0.05 Indicate statistically significant variable; 1. Reference group

## Discussion

This hospital-based cross-sectional study measured the proportion of adherence to dietary practice among diabetes patients. Findings indicate that more than half of participants were not adhering to the recommended dietary practices. Adherence to recommended dietary practices for diabetic patients is very critical to achieve optimal metabolic control, as non-adherence is associated with higher glucose level and cholesterol levels, which eventually lead to major complications [19]. It is widely accepted that diabetes and its complications pose a major burden worldwide and present significant challenges to patients, health care systems and national economies [20]. Overall, 44.3% of participants adhered to dietary recommendations. The degree of adherence identified in this study is relatively lower in comparison to other similar studies [21-24]. This implies the need for sustained effort promoting dietary management for diabetes patients. However, this rate is relatively higher than that found in studies conducted in Mexico, Nepal and India [25, 26]. This discrepancy could be due to variation in the study population, sample size and adherence measurement tool. In this study, the two major reasons cited as barrier to adhere for recommended dietary practice were high frequency of gathering with family and friends and eating out. The same finding has also been reported by other studies conducted in India and Botswana [21, 27]. This could be due the difficult nature of going against the social expectations or pressures of eating with friends and family or the necessity of eating out.

Therefore, caregivers have to play an important role by potentially providing continuous consultation and follow-up regarding the dietary habits of their diabetic patients. Receiving diabetic nutrition was one of the factors significantly associated with adherence to the recommended dietary practice among participating patients. Studies conducted in Ethiopia and South Africa have also stated that diabetic nutrition education had a significant impact on dietary adherence [22, 28]. This might be due to the fact that patients who have received intensive education are more likely to have increased knowledge about the benefit of food management in diabetic control. In addition, patients who have received nutrition education may perceive greater seriousness of not adhering to the recommended dietary regimen. Patients who were diagnosed more than 10 years ago had better adherence to the dietary practice. Several other studies have also revealed a positive association between longer duration of the disease and better diabetic treatment adherence [29-34]. The possible explanation is that patients with longer duration of diabetes will have more frequent contacts with health professionals and are more likely to be given repetitive instruction on treatment adherence, thus, become aware of the acute and chronic complications of uncontrolled blood glucose that eventually leads to adoption of healthy behaviors'.

**Limitations**: despite extensive efforts have been made to minimize possible shortcoming of this study, the finding of this survey will be interpreted in the presence of the following inevitable limitations. The cross-sectional nature of this study makes it unreliable to form a causal relationship between the exposure and the outcome variable. Response biases, mainly the intention of the participants not to respond the actual adherence questions is likely (social desirability bias).

## Conclusion

The prevalence of non-adherence to dietary practice was observed in more than fifty percent of patients, which indicates a major public health problem. Attending diabetic nutrition education and being diagnosed more than 10 years ago were associated with greater adherence to dietary recommendation. All stakeholders including clinicians, dietitians, health educators and policymakers should be made aware about the alarmingly low proportion of adherence to dietary recommendations among the diabetic population in the study area. Therefore, it is important to design strategies to help patients understand their dietary regimens in order to improve their adherence. Otherwise complications of diabetes mellitus, which are debilitating, can increase the burden of a disease that is already increasing. Similarly, large scale studies, particularly with prospective designs, should be undertaken to contribute more information regarding the level of adherence and determinants of non-adherence to diet.

### What is known about this topic

- Studies conducted in lower and middle income countries on diabetes self-care behaviors reported unsatisfactory level of adherence to dietary practice. There was also a report of significant association between diabetic nutrition education and adherence to dietary recommendation in different studies.

### What this study adds

- Significant proportion of non-adherence to dietary recommendation among diabetes patients was observed in spite of numerous efforts spent by stake holders;
- There was a statistically significant association between duration of diabetes and adherence to dietary regimen.

## Competing interests

The authors declare no competing interests.
